# How to optimize the treatment of early stage Parkinson’s disease

**DOI:** 10.1186/2047-9158-4-4

**Published:** 2015-02-25

**Authors:** Fabrizio Stocchi, Laura Vacca, Fabiana G Radicati

**Affiliations:** Department of Neurology, Institute for Research and Medical Care, IRCCS San Raffaele, via della Pisana 235, 00163 Rome, Italy

## Abstract

The approach to early Parkinson’s disease denotes the communication of the diagnosis and important decisions, such as when and how to start treatment. Evidence based medicine and guidelines indicate which drugs have robust evidence of efficacy and tolerability in this specific population. However, *de-novo* patients may show different characteristics and they may be in a different phase of their disease.

In this review, we will give an insight into the appropriate time therapy should be started and the actual knowledge about disease modification therapies. Moreover, the drugs indicated for early treatment will be considered and an indication for the use of these drugs will be given with the support of the actual knowledge.

## Introduction

The approach to early Parkinson’s disease denotes the communication of the diagnosis and important decisions, such as when and how to start treatment. Evidence based medicine and guidelines indicate which drugs have robust evidence of efficacy and tolerability in this specific population. However, *de-novo* patients may show different characteristics and they may be in a different phase of their disease.

The treatment of patients with early Parkinson’s disease should aim to slow down clinical progression, control motor and non-motor symptoms, maintain functioning in daily-life activities, prevent motor complications and minimize the risk of side effects.

In this review the drugs indicated for early treatment will be considered and an indication for the use of these drugs will be given with the support of the actual knowledge.

### When to start treatment

Parkinson’s disease (PD) is a progressive neurodegenerative disorder that is manifested clinically by a resting tremor, rigidity and bradykinesia. These typical motor symptoms are due to the degeneration and loss of dopaminergic neurones in the substantia nigra with consequent reduction in the ability of the brain to form, store and regulate the release of dopamine, which is essential for the control of motor function [1].

The rate of disease progression varies in the early stages, being slower in the less affected patients. In the placebo cohort of the ADAGIO trial, a significant correlation between rate of progression and baseline UPDRS score was found. Patients in the placebo group with the highest quartile of baseline total UPDRS scores (>25 · 5; n = 145) had the greatest rate of progression (change from baseline to 36 weeks of 6 · 0 units [SD 8 · 4]) with a rate of decline of about 9 UPDRS points per year. In contrast, patients with the lowest quartile of baseline UPDRS scores (≤14; n = 160) deteriorated between baseline and week 36 by 2 · 5 units (SD 4 · 8), with an extrapolated rate of deterioration of about 4 units per year. The difference in the progression from baseline to week 36 (last observed value) between the two quartiles was significant (mean difference -3 · 46 [SE 0 · 77]; p < 0 · 0001) [2]. Thus the early period after diagnosis is critical in terms of rate of progression, but it is also in the early stage of the disease that an intervention able to modify the natural course of the disease may be more successful.

Today there is a large debate about the opportunity to start pharmacological treatment as soon as the disease manifests.

Until now, the recommendation that drug treatment should be delayed until the symptoms of PD significantly limited the patient’s motor functions has become established in teaching and part of many guidelines. The rationale for this was to protect the patients from unnecessary side effects, particularly the motor complications associated with levodopa. Moreover, a view also evolved that patients delaying the introduction of pharmacological treatment would respond for longer when the drugs were introduced. Despite the fact that there is no evidence supporting this theory, the majority of clinicians follow it.

Bearing in mind that the denervation in PD begins approximately 6 years before the appearance of symptoms, basal ganglia have a remarkable capacity to cope with progressively low levels of dopamine activating compensatory mechanisms. The appearance of symptoms indicates the point of failure to deal adequately with dopamine depletion. Recently, A. Schapira and J. Obeso proposed that the early restoration of basal ganglia physiology would support the compensatory events and delay the irreversible modification of circuitry that characterizes the clinical progression of PD [3]. However, the theory of an early compensatory effect of symptomatic drug with an associated better long-term symptom control is fascinating and we believe it is time to reconsider the traditional view of starting symptomatic treatment as late as possible.

### Slowing down clinical progression: where we are

While there have been many promising candidate agents based on laboratory studies and pathologic findings, no treatment has as yet been established to have neuroprotective or disease-modifying properties in PD. Several obstacles have been identified that impede the achievement of this goal [4].

### The cause of PD

A number of pathogenic factors have been implicated, including oxidative stress, mitochondrial dysfunction, inflammation, excitotoxicity, and signals mediating an apoptosis cascade [1, 5]. Several environmental factors have been identified as risk factors, as well as a number of gene mutations [6–8] but none these factors seem to be determinant in sporadic cases. At present, it seems likely that sporadic cases are due to a complex interaction between environmental, genetic and epigenetic factors [9].

### Animal model

The 6-OHDA rodent, MPTP mouse and primate models of PD [10, 11] are caused by the acute administration of toxins that likely do not reflect the etio-pathogenesis of PD; they do not accurately reflect the pathologic distribution of the disease. Transgenic models based on genetic causes of PD are more promising [12].

### Clinical trials target validation, scales and biomarkers

If a drug is promising in the laboratory, it ultimately has to be tested in PD patients. This also presents many obstacles that need to be overcome in identifying a neuroprotective agent for PD.

There are few targets that can be assessed that reflect a pathogenic mechanism and all too often one has to proceed without this information. Problems are further confounded by the scales that are currently employed in clinical trials, which have a limited range and are particularly insensitive to detecting change in the early stages of the disease. In particular, the UPDRS scale has a clear “floor effect”, which limits the possibility of measuring improvement in an early, mildly affected population. These problems could be resolved by the development of a validated biomarker that could be used to confirm the diagnosis or to serve as an endpoint to objectively measure disease progression and drug efficacy. Unfortunately, no such biomarker currently exists [13, 14].

### Clinical trial design

No drug has yet been established to have a neuroprotective effect in PD. Several clinical trials of putative neuroprotective agents have shown positive results; however, it could not be determined with certainty if the benefit was due to neuroprotection resulting from potentially confounding pharmacologic or regulatory effects of the study agent [1]. In an attempt to separate an early symptom from a disease modifying effect, the delayed washout and delayed start studies have been proposed [15]. The delayed washout has not been considered for use in PD because of the ethical and practical issues involved in withdrawing therapy from PD patients, particularly for the periods of time necessary to conduct the trial.

The delayed start study [16, 17] was employed in the recent ADAGIO study to try and determine if rasagiline had neuroprotective effects in PD [18]. Rasagiline 1 mg per day met all three prescribed primary endpoints consistent with the drug having a disease-modifying effect. However, the 2 mg dose failed to show a difference between early and delayed treatment at the end of period 2. Thus, the results of the study were inconclusive and further studies testing these doses separately are required to determine which of these results is valid. While the results of ADAGIO are not definitive, the study design does provide a method for differentiating early symptomatic and disease-modifying effects and should facilitate the investigation of new agents. Another approach is the long-term simple study, where subjects are randomised to active treatment or placebo, and then followed for a prolonged period of time (many years) in which the physician can manage the patient in any way they deem to be appropriate. The outcome measure captures factors related to the development of cumulative disability, such as falling, freezing and dementia in addition to standard UPDRS scores [19]. A combination of the delayed start and long-term simple studies offers assessments of mechanism and clinical significance, and provides a roadmap for the development of a neuroprotective drug [20].

### The future

Fortunately, the outlook is improving and there are promising new candidate target drugs based on genetic causes of PD. There are new animal models based on gene mutations associated with familial forms of PD that will likely prove more reliable in predicting the results of promising new therapies in clinical trials. The use of new study designs, such as the delayed start and long-term simple studies provide a roadmap for defining a disease-modifying drug and establishing its clinical significance. Additionally, new clinical trial methodologies designed to reduce variability in data and analytic approaches, such as adaptive design, have facilitated the more rapid and accurate determination of success or failure of a study intervention in both learning and confirming trials.

### Treatment of early disease

#### Levodopa

Levodopa is the gold standard for the treatment of PD, and no medical or surgical therapy has been shown to provide superior anti-parkinsonian benefits than can be achieved with levodopa. However, chronic levodopa treatment is associated with the development of motor complications and non-motor complications in the majority of patients [21]. Unfortunately, it is not still established how levodopa should be administered in the early stage (dose, formulation, interval between doses) in order to minimize the risk of complications.

Indeed, several studies have now shown that continuous infusion of levodopa, which provides a more continuous dopaminergic stimulation [22–24] in PD patients who experience motor complications, is associated with a reduction in both off time and dyskinesia [25–27]. Accordingly, there has been a search for an oral levodopa treatment strategy that might mirror the pharmacokinetics of a levodopa infusion and provide the benefits of the medication without motor complications.

Entacapone is an inhibitor of catechol-O-methyl transferase (COMT) that extends the elimination half-life of levodopa [28], and thus has the potential to provide more continuous availability of levodopa [29]. Based on these considerations, the possibility that levodopa/carbidopa combined with entacapone (LCE), administered 4 times daily at 3.5 hour intervals, might reduce the risk of dyskinesia compared with levodopa/carbidopa (LC) alone was tested in a prospective, double-blind trial; this included 745 subjects treated for 134–208 weeks (the STRIDE-PD study) [30]. The study failed to demonstrate the expected benefit. Indeed, patients randomised to receive LCE experienced an increased frequency of dyskinesia in comparison to patients receiving LC alone. Despite this, we believe the rationale behind the STRIDE-PD remains solid and there are different explanations for the study failure. The four daily doses administered every 3.5 hours do not provide a stable and continuous levodopa plasma level, even when administered with entacapone. It is difficult to obtain a good compliance from early non-fluctuating patients in terms of exact dose timing. Daily levodopa load was higher in the entacapone–levodopa group.

While the study failed to meet its primary endpoint, the data from the STRIDE-PD study were explored to assess the role of levodopa dose and other risk factors in the development of dyskinesia and wearing-off in this relatively long-term, double-blind trial [31]. This has great importance, as there has been a paucity of double-blind clinical trials informing the optimal method for introducing levodopa to PD patients.

Patients were divided into 4 dose groups based on nominal levodopa dose at time of onset of dyskinesia (or study conclusion if no dyskinesia): Group 1, <400 mg/day (n = 157); Group 2, 400 mg/day (n = 310); Group 3, >400–600 mg/day (n = 201); Group 4, >600 mg/day (n = 77). Similar analyses were performed with respect to wearing-off. Time to onset and frequency of dyskinesia/wearing-off were compared using Cox proportional hazards model. A stepwise Cox proportional hazards model was used to screen predictive factors in a multivariate analysis. The risk of dyskinesia in the total population was increased in a levodopa dose-dependent manner (p < 0.001). Analysis using levodopa equivalent doses showed comparable results. A stepwise cox proportional hazards model was used to screen predictive factors for the emergence of dyskinesia in a multivariate analysis. Young age at onset of PD was the most important single explanatory factor. The next most important factor was the nominal levodopa dose. These factors were followed by: lower weight, region (North America), treatment allocation (LCE), female gender and baseline UPDRS part II score. Risk of wearing-off also increased in a levodopa dose-dependent manner (p < 0.001). Multivariate analyses showed similar predictors as dyskinesia, but included baseline UPDRS III, and excluded weight and treatment allocation.

This study reinforced the concept that the risk of developing dyskinesia and wearing-off each increased with higher levodopa doses. Thus physicians should use the lowest dose of levodopa that will provide satisfactory clinical control to minimize the risk of dyskinesia and wearing-off [31].

### Dopamine agonists

Dopamine agonists (DAs) have longer plasma elimination half-lives than levodopa. Their use has therefore been considered an opportunity to improve continuous drug delivery. By the late 1980s, ergot-derived DAs (bromocriptine, pergolide, lisuride, and cabergoline) already had an established role as adjuncts to levodopa in advanced PD because of their ability to reduce “off” time and, in many instances, allow a lowering of the levodopa dose, reducing dyskinesias. Later, the use of oral DAs has moved to earlier stages of PD [31]. Following early pilot uncontrolled observations in PD patients [32–34], the first levodopa-controlled trials were published in the mid-1980s, suggesting that patients starting on an agonist had a lower risk of subsequent motor complications than those starting on levodopa [35–40]. This consistent finding thereby changed the standard of care from the primary use of levodopa to the primary and early use of DAs, especially in younger-onset patients. By the late 1990s, ergot DAs had been largely replaced by non-ergot drugs like ropinirole and pramipexole because of reports of fibrotic adverse reactions, including cardiac valvular damage from pergolide and cabergoline [41–43]. Recently, controlled-release formulations of oral ropinirole [44, 45], pramipexole [46] and transdermal formulations of rotigotine have been developed [47, 48], offering the convenience of a once-daily regimen.

After more than a decade of widespread use of DAs in early PD, long-term clinical experience has taught us that the initial innovative and attractive finding of delaying time to dyskinesia with a DA is far from being the sole relevant outcome to consider. Indeed, our knowledge has extended to several other important issues:

- The large-scale use of DAs revealed that these drugs carry a greater risk than levodopa of previously unknown or underestimated and potentially troublesome adverse drug reactions, including abnormal daytime somnolence [49–52], leg oedema [53], and impulse control disorders [54–56].
- Levodopa must almost inevitably be added to a DA to keep control of parkinsonism after a few years of treatment, although the early use of a DA reduces the cumulative dose of levodopa and explains the long-term benefit of DAs on dyskinesia [57–59].
- The long-term disability and quality of life of patients initially randomised to an agonist or levodopa do not differ [60], although the impact of dyskinesia on quality of life can vary greatly from patient to patient.
- It is now realized that in most PD patients long-term disability (15 years) is driven by problems, such as falls or dementia, and these are not influenced by early treatment with agonists or from other drugs [61].
- Finally, it is also now agreed that there is no clinical evidence supporting the theoretical rationale to avoid levodopa because of its potential oxidative “toxicity” toward dopamine neurons [62]. However, it is recommended to use low doses of levodopa to avoid motor complications.

Dopamine agonists remain important drugs in the physician’s armamentarium; however, pros and cons of their use should be considered and the inappropriate “levodopa phobia”, raised especially among patients [63], should be reconsidered. DA agonists can be used as first line treatment but the dose should be carefully evaluated. Levodopa can be added to a DA agonist when patients require it, offering the possibility of giving lower doses of both drugs. Certainly DA agonists can be added to levodopa instead of increasing the levodopa dose, which provides further advantages for symptoms that respond better to dopamine agonists (i.e. depression, apathy) and for reducing the risk of dyskinesia [59].

### MAO-B inhibitors

Selegiline (deprenyl) was the first selective, irreversible inhibitor of monoamine oxidase type B (MAO-B) used in the treatment of Parkinson’s disease. Because of its capacity for interfering with oxidative stress and for blocking MPTP toxicity [64], selegiline was tested in the first major trial as a putative disease-modifying agent. The Deprenyl and Tocopheral Antioxidative Therapy of Parkinsonism trial (DATATOP) was positive in showing a significant delay in the need for levodopa treatment in patients treated with deprenyl versus placebo [65]; it also illustrated that a mild symptomatic effect in a trial such as this would confound any neuroprotective interpretation [66]. However, the DATATOP study consolidated the use of selegiline in the treatment of early PD. The amphetamine-like metabolites of selegiline raised some concerns about its safety and mortality was reportedly increased when selegiline and levodopa were given together, in comparison to treatment with levodopa alone. However, a large meta-analysis of 5 long-term studies and 4 separate studies did not support this conclusion. Selegiline seems to be generally well tolerated in combination with other drugs [67].

Rasagiline is another MAO-B inhibitor, with different metabolites than selegiline, to be successfully developed for PD therapy. From a symptomatic perspective, rasagiline 1 mg/day proved to be efficacious as monotherapy in early PD [68, 69]. The good tolerability of rasagiline and its ease of use (1 dose, once daily, no titration) makes this drug an appealing option to start therapy in PD [70]. The current popularity of rasagiline is also related to the finding that the drug has neuroprotective properties in vitro [71, 72] and was the first putative disease-modifying agent to be tested with a randomised delayed-start design in PD [18]. Recently, a pragmatic, open label randomised trial has been published to evaluate, out of three classes of drug (levodopa, dopamine agonists, or monoamine oxidase type B inhibitors (MAOBI)) for initial treatment, which of these provides the most effective long-term control of symptoms and best quality of life for people with early Parkinson’s disease [73]. The authors found a very small but persistent benefit for patient-rated mobility scores when treatment was initiated with levodopa compared with levodopa-sparing therapy. Furthermore, MAOBI as initial levodopa-sparing therapy was at least as effective as dopamine agonists. Recently, in a double-blind control study (ANDANTE study) [74] rasagiline 1 mg/d provided statistically significant improvement when added to dopamine agonist therapy and was well tolerated. This demonstrates that a combination of DA-agonists and MAOB inhibitor may offer a better control of symptoms than monotherapy.

### Practical considerations

A patient is defined *de novo* when he or she has never been exposed to specific pharmacological treatment. The definition does not imply the severity of the disease and even in clinical trials on an early population there are differences in the severity of the population participating in the study. In the ADAGIO trial, for example, the mean UPDRS-total score at baseline was 20.4 (8.5); whereas in the TEMPO study with the same population, the UPDRS-total score at baseline was 25.0 (10.8). Within this population there is a very large range of severity (see above). In real life, the majority of patients receive the diagnosis 1–2 years after the appearance of the first motor symptom. Sometimes the patients are not treated at the first instance but only when the disease progresses. These variables lead to the evidence that the *de novo* population is very heterogeneous and the decision about the drug initially has to take into account the general characteristics of the patient, such as age, cognitive status, comorbidities, occupation, and the most affected side (dominant or non-dominant) but also of the severity of the symptoms and the presence of non-motor symptoms. For example, fatigue is a very common symptom of PD [75] and in a recent double-blind study on an early *de novo* population, rasagiline significantly improved fatigue [76].

Guidelines refer to *de novo* population in general and sometimes take into account the age but not other determining factors such as severity. *De novo* patients can be classified as mild, moderate or severe; mild being a patient with UPDRS ≥20 and HY stage 1, moderate UPDRS 20–30 HY stage 1–2, severe UPDRS >30 HY 2 or more. Additional important aspects are the most affected side, age and metal status. Combining this information, along with the efficacy and safety characteristics of each drug, the physician can make the right choice and possibly predict the induced benefit.

Today we may also consider a combination of drugs rather than using a single drug at high dose. Rasagiline can be successfully combined with DA-agonists and L-dopa, and L-dopa can be combined with a DA and MAOB. A combination of drugs may be more or equally effective but may also reduce the dose of each of them, which minimizes the risks of side effects.
